# A dataset with complete geographic distributions of eight zonal monospecific forest types in mainland Spain

**DOI:** 10.1016/j.dib.2020.106681

**Published:** 2020-12-26

**Authors:** Rut Sánchez de Dios, Maria E. Sanjuán, Helios Sainz, Alberto Ruiz, Jaime Martínez Valderrama, Gabriel del Barrio

**Affiliations:** a Departamento de Biodiversidad, Ecología y Evolución, Universidad Complutense de Madrid, 12 José Antonio Novais St., 28040 Madrid, Spain; b Estación Experimental de Zonas Áridas, CSIC, Sacramento Rd., 04120 La Cañada de San Urbano, Almería, Spain; c Departamento de Biología, Universidad Autónoma de Madrid, Darwin St., Cantoblanco, 28049 Madrid, Spain

**Keywords:** European Union nature information system, Korcak exponent, Natura 2000 network, Segmented regression

## Abstract

Distribution area and surface are both parameters of paramount importance for habitat management, monitoring and conservation. Here we present the distribution of eight zonal forest types of mainland Spain that are consistent with the Habitat Types (HT) listed in Annex I of the European Union Habitats Directive 92/43 EC. Their dominant species and HT codes are *Fagus sylvatica* (9120, 9130 and 9150), *Quercus robur* and *Q. pyrenaica* (9230), *Q. suber* (9330), *Pinus uncinata* (9430), *P. nigra* ssp. *salzmannii* (9530) and *P. pinea* (subset of 9540). These distributions are based on tesserae from the 1:50,000 Spanish Forest Map and are the result of sorting assisted by supplementary databases. The distributions are presented as vector coverages, and provide three information levels of increasing detail: geographic distribution, basic forest type and structural forest patch. Two R scripts are also included with the dataset. They implement a segmented regression approach to investigate forest fragmentation on these or other patch-like data.

## Specifications Table

**Table:** 

Subject	Nature and Landscape Conservation
Specific subject area	Biogeography and spatial distribution of habitat types
Type of data	Geospatial: vector coverages in shapefile format
How data were acquired	1) Basic forest types found by progressive sorting of two implementations of the 1:50,000 scale Mapa Forestal de España [Spanish Forest Map] MFE50, supplemented by other databases. 2) Such basic types aggregated to Habitat Types meeting the definitions in the EU Habitat Directive, Annex 1.
Data format	Filtered (see above entry and References for the raw data)
Parameters for data collection	Monospecific forest types: tree canopy density over 10% and dominant species prevalence over 70%
Description of data collection	Each coverage corresponds to a EU Habitat Directive Habitat Type. Its attribute files include the following specific fields for each polygon: individual ID, ID in MFE-50 (which links with that database), basic forest type, Habitat Type, European Forest Type and EUNIS level 3 code.
Data source location	1) Mapa Forestal de España 1:50.000 (MFE50) Ministerio de Agricultura, Alimentación y Medio Ambiente Madrid, Spain.https://www.miteco.gob.es/es/biodiversidad/servicios/banco-datos-naturaleza/informacion-disponible/mfe50.aspx 2) Information System of Forest Tree Species (GIS-FOREST) Instituto Nacional de Investigación y Tecnología Agraria y Alimentaria Madrid, Spain.https://sites.google.com/site/sigtreeforestspeciesenglis/
Data accessibility	Repository name: Distribution of eight forest habitats of European Interest in mainland Spain, and R code for Korcak fragmentation analysis Data identification number: DOI: 10.17632/bm59f4nddy.4 Direct URL to data: http://dx.doi.org/10.17632/bm59f4nddy.4
Related research article	Gabriel del Barrio, Helios Sainz, Maria E. Sanjuán, Rut Sánchez de Dios, Jaime Martinez Valderrama and Alberto Ruiz. Abrupt fragmentation thresholds of eight zonal forest types in mainland Spain. Forest Ecology and Management. https://doi.org/10.1016/j.foreco.2020.118788

## Value of the Data

• The distributions found are crucial for ecological studies dealing with the forest types concerned.
• Potential users include ecologists fitting predictive distribution models to investigate climate and land use change, foresters working at the patch level and conservation practitioners seeking to optimize conservation networks.
• The fine-scale patches provided enable studies on spatial structure and fragmentation of these forest types.
• Beyond simply indicating the geographic distributions of these European Habitat Types, this dataset adds two higher levels of detail: basic forest types and structural forest patches.

## 1 Data Description

This dataset consists of complete distributions observed at the 1:50,000 scale of eight zonal forest types of mainland Spain. These distributions are built up from explicitly identified forest patches.

These forest types are consistent with Habitat Types (HT) listed in Annex I of the European Union Habitats Directive 92/43 EC [1]. More details about them are available in the reference interpretation manual [2]. Their HT codes and names are shown in Table 1.

**Table 1:** Summary of the zonal forest types included in the database.

Dominant species	HT code	HT name	Basic forest types*	Total number of patches	Total Surface (ha)
*Fagus sylvatica*	9120	Atlantic acidophilous beech forests with *Ilex* and sometimes also *Taxus* in the shrublayer (*Quercion robori-petraeae* or *Ilici-Fagenion*)	232	874	63,687
*Fagus sylvatica*	9130	*Asperulo-Fagetum* beech forests	231	516	49,415
*Fagus sylvatica*	9150	Medio-European limestone beech forests of the *Cephalanthero-Fagion*	234, 235	1514	41,999
*Quercus pyrenaica*	9230	Galicio-Portuguese oak woods with *Quercus robur* and *Quercus pyrenaica*	321, 322, 323, 324	16,498	750,076
*Quercus suber*	9330	*Quercus suber* forests	430, 431, 432, 433, 434	3754	221,818
*Pinus uncinata*	9430	Subalpine and montane *Pinus uncinata* forests	111, 112, 113, 114	2671	89,497
*Pinus nigra*	9530	(Sub-) Mediterranean pine forests with endemic black pines	511, 512, 513, 514	5267	378,195
*Pinus pinea*	9540	Mediterranean pine forests with endemic Mesogean pines	631, 632, 633	2448	131,890

* See details in [3].

Distributions are provided as vector coverages in shapefile format, which can be directly imported by most Geographic Information Systems (GIS) software. Each coverage is labeled with its HT code, and consists of five files: three mandatory ones (.shp, .shx and .dbf, respectively conveying feature geometry, positional index and attribute table), and two optional (.prj and .qpj, both containing the projection description). The coordinate reference system is EPSG 25830: datum ETRS89 and projection UTM zone 30 N.

Coverages features are polygons corresponding to forest patches, which in turn, correspond to tesserae in the Spanish Forest Map (MFE50) [4]. The attribute table contains the following fields:

• OBJECTID: individual polygon identification number;
• POLIGON: polygon identification number in the source database (MFE-50);
• S_NAT: Basic forest type code (specific to this dataset, see next section);
• HT: Habitat Type (Annex I of the European Union Habitats Directive 92/43 EC);
• EFT: European Forest Type (European Environment Agency);
• EUNIS_3: EUNIS habitat classification, level 3 (European Environment Agency);
• SHAPE_LENG: polygon perimeter (meters)
• SHAPE_AREA: polygon area (square meters).

The attribute table can be linked to the MFE50 source database (through the link field POLIGON), to check other forestry attributes of each polygon. The fields EFT [5] and EUNIS_3 [6] enable wider harmonisation and use for EU level mapping efforts and projects.

In addition to coverages, two scripts in R language [7] are provided with the dataset. These correspond to the fragmentation analyses made in the related research article: determination of the Korcak exponent by segmented regression. This is a method to determine patchiness by exploring patch frequency as a function of patch size. The novelty here is that such an exploration is done by size intervals, leading to the identification of area thresholds. Both scripts can be easily adapted for equivalent purposes according to user needs by following the instructions in the annotated headings. Whilst the scripts develop insights on the use of coverages, they are not required if used for other purposes.

## 2 Experimental Design, Materials and Methods

The cartographic base for this dataset was the MFE50. It is a comprehensive mapping, made by photointerpretation and fieldwork, of forest and shrub lands of Spain. Its tesserae are associated with distinct forest patches and have fields describing total and only-tree canopy density, structural vegetation type, spatial grouping of vegetation, up to three dominant species and their cover density, land use, etc. However, whilst complete structural information is readily available in the MFE50, this product cannot be used directly to depict the distribution of forest HTs. The dataset presented here is an evolution of the MFE50 to fill precisely this need.

In a first step, forest tesserae were separated from scrub, pasture or *dehesas*. Then, all forest tesserae were sorted, retaining only those with a tree canopy cover of over 10% [8]. After that, monospecific forests were differentiated from mixed forests [9,10] by selecting only the tesserae where the occupation of the dominant species was over 70%. Afforestation and *dehesas* were distinguished from the monospecific forests using the afforestation maps in the Spanish Information System of Forest Tree Species [11] in addition to the information provided by the MFE50 itself, and discarded. Finally, all remaining tesserae were assigned to a basic forest type following the classification by Sainz Ollero and Sánchez de Dios [12]. Additional national and regional lithological, geomorphic and vegetation maps were also used for this [3]. The basic forest types were then grouped into HTs.

That process resulted in 24 basic forest types grouped into the eight HTs presented in this article (Table 1). The data are comprehensive for each HT except in the case of 9540, which includes only *Pinus pinea* forests. This cartography adds ecological value with respect to the sources used, because it combines the species distribution from MFE 1:50,000 with environmental and biogeographic variables on climate, substrate and altitudinal vegetation belts.

It is worth mentioning that this dataset was a benchmark exercise done within the framework of a Spanish Forest Administration project, for setting up a nationwide Spanish habitat surveillance and monitoring system. The overall outcome is expected to contain 78 monospecific and 32 mixed forest types for all of Spain, grouped into 22 EUNIS HTs. More information can be found in [3], within the project website [13].

## CRediT Author Statement

**Rut Sánchez de Dios:** Validation, Investigation, Writing—original draft preparation, Writing—review and editing, Data curation. **María E. Sanjuán:** Methodology, Validation, Data curation. **Helios Sainz:** Validation, Investigation, Writing—original draft preparation, Supervision, Project administration, Funding acquisition. **Alberto Ruiz:** Software, Resources, Visualization. **Jaime Martínez-Valderrama:** Investigation, Resources. **Gabriel del Barrio:** Conceptualization, Formal analysis, Writing—original draft preparation, Writing—review and editing, Supervision, Project administration, Funding acquisition.

## Declaration of Competing Interest

The authors declare that they have no known competing financial interests or personal relationships which have, or could be perceived to have, influenced the work reported in this article.
